# Dynll1-PI31 Interaction Enhances Proteolysis Through the Proteasome, Representing a Novel Therapeutic Target for INF2-Related FSGS

**DOI:** 10.34067/KID.0000000659

**Published:** 2024-12-02

**Authors:** Jillian Williquett, Chandra Perez-Gill, Chantal Allamargot, Faith Rooney, Martin R. Pollak, Hua Sun

**Affiliations:** 1 Division of Nephrology, Stead Family Department of Pediatrics, Carver College of Medicine, The University of Iowa, Iowa City, Iowa; 2 Department of Pathology, University of Colorado Anschutz Medical Campus, Aurora, Colorado; 3 Central Microscopy Research Facility, Office of the University of Iowa Vice President for Research, The University of Iowa, Iowa City, Iowa; 4 Beth-Israel Deaconess Medical Center, Harvard Medical School, Boston, Massachusetts

**Keywords:** cell and transport physiology, familial nephropathy, glomerulosclerosis, molecular biology, podocyte, transgenic mouse

## Abstract

**Key Points:**

- The R218Q mutation disrupts sequestration of Dynll1 by inverted formin 2, promotes Dynll1-PI31 interaction, and enhances proteasome-mediated nephrin degradation.
- Suppression of proteasome-mediated proteolysis with proteasome inhibitors is a new therapeutic strategy for inverted formin 2-mediated FSGS.

**Background:**

The p.Arg218Gln (R218Q) mutation in the inverted formin 2 (*INF2*) gene causes podocytopathy prone to FSGS. This mutation disrupts the ability of INF2 to sequester dynein light chain 1 (DYNLL1), thus promoting dynein-mediated mistrafficking of the slit diaphragm protein, nephrin, to proteolytic pathways. Bortezomib, a proteasome inhibitor, stabilizes nephrin in R218Q knockin (KI) podocytes, suggesting a role for the ubiquitin proteasome system (UPS) in dynein-driven pathogenesis. However, the link between dynein and the UPS is unknown. This study tested the hypothesis that INF2 R218Q promotes proteasome-mediated degradation of nephrin through an increased interaction between Dynll1 and the proteasomal inhibitor of 31kD (PI31), a Dynll1 adaptor that potentially couples the UPS with dynein cargoes.

**Methods:**

The essential role of PI31 in UPS-mediated degradation of nephrin, a known dynein cargo, was studied in cultured R218Q KI mouse podocytes by applying genetic or chemical interventions to inhibit the activity of PI31 or of the proteasome. The protective effect of bortezomib in dynein-driven podocytopathy and FSGS was tested in R218Q KI mice challenged with puromycin aminonucleoside, a murine model of FSGS.

**Results:**

The R218Q mutation in INF2 disrupted sequestration of Dynll1 by INF2, allowing Dynll1 to be captured by PI31 and promoting dynein-mediated transport of nephrin to the proteasome. Each of the following manipulations was sufficient to restore nephrin proteostasis in R218Q KI podocytes: knocking down *Dynll1* or *PI31*, inactivating dynein, or inhibiting the activity of the proteasome. In R218Q KI mice challenged with puromycin aminonucleoside, dynein-mediated mistrafficking and depletion of nephrin were correlated with increased Dynll1-PI31 interaction; the resulting podocytopathy and FSGS were ameliorated by bortezomib.

**Conclusions:**

The Dynll1-PI31 interaction facilitates dynein-driven trafficking of nephrin to the proteasome and proteasome-mediated degradation of nephrin in INF2-R218Q-mediated podocytopathy. This mechanism offers new therapeutic strategies for INF2-related FSGS by using pharmacologically available proteasome inhibitors.

## Visual Abstract

**Figure:**
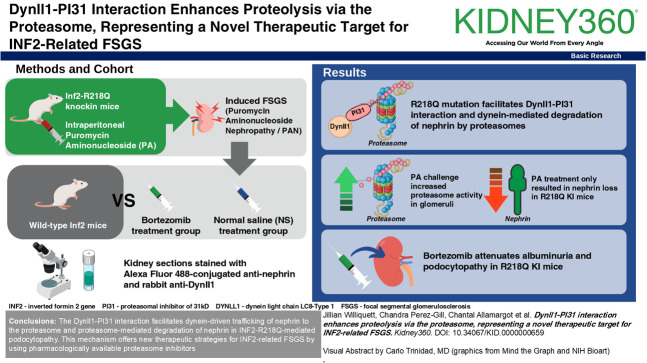

## Introduction

The inverted formin 2 (*INF2*) gene encodes a formin family protein that is highly and selectively expressed in podocytes. Formins are a multigene family of proteins with diverse abilities to regulate cellular functions involving actin and microtubule cytoskeletal networks.^1–4^ Mutations in *INF2* are a major cause of familial and sporadic cases of autosomal dominant FSGS, as well as non-FSGS types of podocytopathy with poor medical response and clinical prognosis.^5–7^ Despite many studies investigating the mechanism of *INF2* mutations in causing FSGS,^4,8–12^ the mainstay of therapy, renin-angiotensin II-aldosterone blockage, has not improved the unfavorable outcome of the disease. Therefore, mechanistic studies to identify more effective therapeutic targets remain of high interest for this challenging genetic podocytopathy.

Our previous study uncovered a new function of INF2 in regulating cytoplasmic dynein, a motor protein complex that mediates retrograde trafficking of membrane-localized proteins along microtubules to cytosolic organelles for processing. We demonstrated that INF2 maintains the quiescent state of dynein by sequestering a key dynein component, dynein light chain 1 (Dynll1); FSGS-causing mutations within the diaphanous inhibition domain of INF2 disrupt the sequestration of Dynll1 by INF2, resulting in enhanced dynein-mediated trafficking of nephrin. Nephrin is a key component of the slit diaphragm (SD) protein complex of podocytes, a complex that constitutes the molecular sieve of the glomerular filtration barrier.^4^ R218Q (c.653G>A, pArg218Gln),^5,7^ one of these representative INF2 mutations that causes podocytopathy, was found to promote dynein-mediated proteolysis by diverting its cargoes (*e.g*., nephrin) to the autophagy lysosome system (ALS).^4^ The ALS and the ubiquitin proteasome system (UPS) are two major proteolytic systems with complimentary roles in maintaining proteostasis in podocytes.^13^ The ALS clears long-lived, large protein/organelle aggregates that are cytotoxic,^14,15^ and the UPS digests short-lived, misfolded proteins through endoplasmic reticulum-associated degradation, for which nephrin is a known substrate.^16^ Recently, our *in vitro* experiments showed that dynein-dependent degradation of nephrin in R218Q knockin (KI) mouse podocytes could be rescued by bortezomib, an inhibitor of the UPS,^17^ suggesting dynein may also sort nephrin for proteasome-mediated degradation (*i.e*., that nephrin can be degraded by both the UPS and the ALS).

A protein-protein interaction screen for binding partners of human proteasomal inhibitor of 31kD protein (PI31) identified Dynein light chains (DYNLL1 and DYNLL2) as PI31-binding partners, distinguished by coimmunoprecipitation followed by mass spectrometry.^18^ PI31, originally named for its ability to inhibit the 20S proteasome in cell-free conditions, facilitates proteasome function in cells through several mechanisms, namely by promoting 26S assembly, competing with proteasome regulators, and transporting proteasomes to dynein cargoes, particularly during neurogenesis.^19–21^ Although upregulated expression of PI31 was found in podocytes of individuals with Fabry lysosomal storage disease,^13^ the role of PI31 in podocyte pathophysiology is poorly understood. Here, motivated by the results of the interaction screen, we tested PI31’s role in connecting dynein and the UPS, and if this connection is affected by INF2 R218Q to induce dynein-mediated depletion of nephrin through the UPS. Furthermore, we elucidated whether targeting the proteasome protects R218Q transgenic mice from developing podocytopathy and FSGS.

## Methods

### Reagents

The antibodies, siRNA duplex sequences, and chemical compounds used in this study are listed in Tables 1–3, respectively.

**Table 1:** Antibodies and assay kits

Antibody	Company	Cat #
Mouse antinephrin (G-8)	Santa Cruz	sc-376522, AF488, AF594
Rabbit antinephrin	Invitrogen	PA5-91907
Mouse anti-Dynll1	Santa Cruz	sc-136287
Rabbit anti-Dynll1	Thermo Fisher	PA5-97920
Rabbit anti-PI31	Sigma	HPA041300
Rabbit anti-INF2	Bethyl Lab	A303–427A
Mouse antiubiquitin	Santa Cruz	Sc-8017
Rabbit anti-WT1	Invitrogen	MA542786
Mouse antisynaptopodin	Invitrogen	MAB8977SP
Mouse anti-*β*-actin-HRP	Santa Cruz	Sc-47778
Albumin creatinine ratio assay kit	Abcam	ab241018
K48 polyubiquitin chain capture assay kit	UBPBio	J4430
CellBrite steady 650 membrane stain	Biotium	30108
20S proteasome activity assay kit	MilliporeSigma	APT280

Cat, catalog number; Dynll1, dynein light chain 1; HRP, horseradish peroxidase; INF2, inverted formin 2; PI31, proteasomal inhibitor of 31kD; WT1, Wilms tumor 1.

**Table 2:** Mouse siRNA duplex sequences

Target	Cat#	Sense (5’-3’)	Antisense (5’-3’)
PI31	sc-152248A	GAC​UUC​CAC​AGG​ACC​UAU​Att	UAU​AGG​UCC​UGU​GGA​AGU​Ctt
PI31	sc-152248B	CCU​GUA​GGC​UAG​UUU​CAU​Att	UAU​GAA​ACU​AGC​CUA​CAG​Gtt
PI31	sc-152248C	CUA​GUA​ACG​ACC​UGU​GAA​Att	UUU​CAC​AGG​UCG​UUA​CUA​Gtt
Dynll1	sc-36229A	GGC​CAU​UCU​UCU​GUU​CAA​Att	UUU​GAA​CAG​AAG​AAU​GGC​Ctt
Dynll1	sc-36229B	CAC​CUC​GUU​UGA​AUC​UGU​Utt	AAC​AGA​UUC​AAA​CGA​GGU​Gtt
Dynll1	sc-36229C	GGC​UUC​AUU​CUC​UGU​ACA​Att	UUG​UAC​AGA​GAA​UGA​AGC​Ctt
Control	sc-37007	UUC​UCC​GAA​CGU​GUC​ACG​Utt	ACG​UGA​CAC​GUU​CGG​AGA​Att

Cat, catalog number; Dynll1, dynein light chain 1; PI31, proteasomal inhibitor of 31kD.

**Table 3:** Chemical compounds

Chemical	CAS #	Company/Cat#	Dose of i.p. Injection in Mice
Bortezomib	179324-69-7	Sigma	0.5 mg/kg twice a week for 4 weeks^26,27^
PA	58-60-6	MedChemExpress	450 mg/kg once^24,25^

Chemical	CAS#	Company/Cat#	*In vitro*
Ciliobrevin D	1370554-01-0	Sigma/250401	50 *µ*M
Bortezomib	179324-69-7	Sigma	100 nM

CAS, chemical abstracts service number; i.p., intraperitoneal injection; PA, puromycin aminonucleoside.

### Podocyte Experiments

Podocytes isolated from wild-type *INF2 (wt)* or *INF2 R218Q (R218Q)* KI mice were conditionally immortalized by expression of a temperature-sensitive mutant of the SV40 Large-T antigen per Saleem’s protocol.^4,22^ Podocytes were maintained in Roswell Park Memorial Institute 1640 media with 10% fetal bovine serum and 1% insulin–transferrin–selenium supplement (Gibco) and differentiated at 37°C for 2 weeks. For siRNA-mediated gene silencing, cells were transfected with siRNA Oligo Duplex targeting *Dynll1* or *PI31* for 72 hours and compared with control cells transfected with a nontargeting siRNA duplex (Table 2). Cell lysates were processed for *immunofluorescent staining, K48 Polyubiquitin Chain Capture assay, proteasome activity assay*, and *western blotting* (Supplemental Methods).

### Nephrin Degradation Assay

We used cycloheximide (CHX) to inhibit new protein synthesis, allowing us to monitor protein turnover. Cells were incubated in media containing 10 *µ*M CHX^23^ for 2 hours, following a 12-hour preincubation with bortezomib (100 nM),^17^ Ciliobrevin D (50 *µ*M),^4,17^ or 0.3% DMSO only (vehicle control). After normalizing nephrin against *β*-actin, the degradation of nephrin was calculated as the reduction of nephrin protein after CHX incubation [(CHX_0h_-CHX_2h)_/CHX_0h_)×100%].

### Transgenic Mice and Animal Experiments

The INF2-R218Q transgenic mice were created by Regeneron Pharmaceuticals, by homologous recombination, per the VelociGene method (mouse strain C57BL/6, ID VG5159/5161).^4^ The R218Q KI mice have no spontaneous podocytopathy, but typical FSGS can be induced by challenging the R218Q KI mice with puromycin aminonucleoside (PA).^4^ We refer to this type of induced FSGS as puromycin aminonucleoside nephropathy (PAN) of the R218Q KI mice, a model representative of human FSGS related to the INF2 R218Q mutation. Three-month-old male littermates of different genotypes (*INF2 wt/wt, wt/ki, ki/ki*) received a single intraperitoneal injection (i.p.) of PA diluted in normal saline (NS), at the dose of 450 mg/kg body weight to induce podocyte injury in R218Q KI mice, but not in the *wt* mice.^4,24,25^ The PA-injected mice were then randomized to a bortezomib treatment group (i.p. injection of bortezomib at the dose of 0.5 mg/kg body weight twice weekly on Mondays and Thursdays for a total of 4 weeks)^26,27^ and to an NS treatment group, with i.p. injection of the bortezomib or the NS starting the day after PA injection. The urine albumin-to-creatinine ratio (UACR) was quantified using the Albumin Creatinine Ratio Assay Kit (Abcam # ab241018). At the end of the 4-week treatment, the mice were anesthetized, and the kidneys were harvested for biochemical (*coimmunoprecipitation, Western blotting,* and *proteasome activity assay* as described in Supplemental Methods) and morphological studies.^4,17^ Mouse glomeruli were isolated by using Dynabeads M-450 Tosylactivated (Invitrogen # 14013) perfusion on the basis of a standard protocol.^28^ Histological and ultrastructural features were quantified and compared among the nine groups of mice (*wt/wt, wt/ki, ki/ki*, each treated under three conditions: no treatment control, PAN+NS, and PAN+Bortizomib), with six mice analyzed per group. The following features were quantified and compared: (*1*) the percentage of glomerulosclerosis per mouse kidney section, (*2*) the average percentage of interstitial fibrosis in three nonoverlapping, low-power views (10×) per mouse kidney section, (*3*) the average number of SDs per *µ*m, and d) the average fraction of capillary loop covered by foot processes without effacement in three nonoverlapping, ultrastructural (2000× magnification) views per mouse kidney section. Kidney sections were costained with mouse Alexa Fluor 488-conjugated antinephrin and rabbit anti-Dynll1 (followed by Alexa Fluor 594-conjugated anti-rabbit IgG, Table 1). The dynein-mediated trafficking of nephrin was quantified as the Dynll1-nephrin colocalization index, which was measured as the Mander's overlap coefficient and as the Pearson correlation coefficient, using the Colo2 plugin of Fiji software.^17^ The mean of measurements in five randomly picked glomeruli per mouse kidney section were calculated for comparison. The overall protein levels and distribution of nephrin were also shown by costaining the kidney sections for nephrin and Wilms tumor 1 (WT1).

### Statistical Analyses

Data analyses were performed using Graphpad Prism 9. Histology and immunofluorescent-signal analysis were performed using National Institutes of Health Fiji/ImageJ software. Data were expressed as the mean±SEM. An independent sample *t* test was used to compare the difference between two groups. One-way ANOVA was used for comparisons among multiple groups, and a *post hoc* q test was used to compare the difference between groups. When analyzing data collected in mice with three genotypes and different treatments, data were analyzed using a two-way ANOVA followed by a Bonferroni *post hoc* test. In a two-tailed test, *P* < 0.05 was considered significant.

### Study Approval

The animal protocol was approved by the Institutional Animal Care and Use Committee of the University of Iowa (#3072340) and is in accordance with the National Institutes of Health guidelines for use of live animals.

## Results

### The R218Q Mutation Facilitates Dynll1-PI31 Interaction and Dynein-Mediated Degradation of Nephrin by Proteasomes

**Figure 1:**
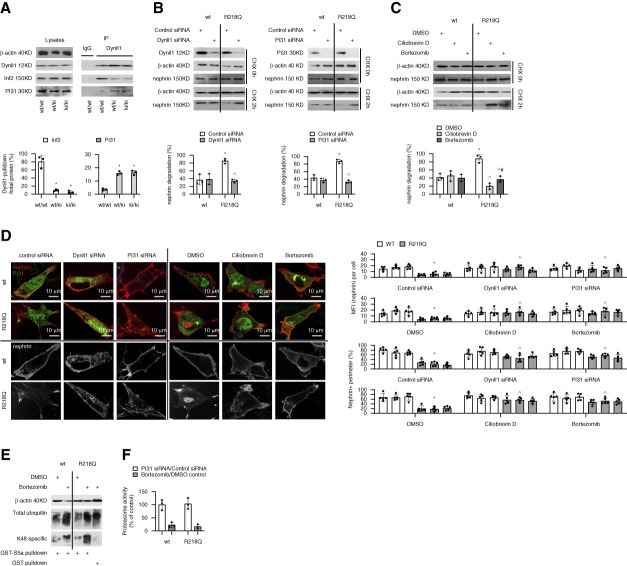
**INF2 R218Q promotes proteasome-mediated degradation of nephrin through Dynll1-PI31 interaction**. (A) Increased endogenous Dynll1-PI31 interaction and corresponding reduced Dynll1-INF2 interaction in glomerular lysates of *R218Q* transgenic mice (*wt/ki and ki/ki* versus *wt/wt*), as measured by Co-IP and quantified as the fraction of INF2 or PI31 protein in the Dynll1-pulldown relative to the total amount in the lysates. *n*=3, * *P* < 0.05 versus *wt/wt*. (B and C) The degradation of nephrin protein in CHX-treated wt and R218Q KI (R218Q) podocytes with siRNA-mediated knockdown of *Dynll1* or *PI31,* compared with cells transfected with control siRNA (B), or in cells treated with Ciliobrevin D (50 *µ*M) or bortezomib (100 nM), compared with cells treated with vehicle (0.3% DMSO; C). After normalizing nephrin to *β*-actin, the percent degradation of nephrin was calculated as ([CHX_0_-CHX_2h_]/CHX_0_)×100%. *n*=3, * *P* < 0.05 versus *wt+control siRNA, ^ P* < 0.05 versus *R218Q+control siRNA*. # *P* < 0.05 versus *R218Q+Ciliobrevin D*. (D) Immunofluorescent staining of nephrin (red) and PI31 (green) in wt or R218Q podocytes with different treatments. The MFI of nephrin staining per podocyte and the percentage of peripheral membrane that was nephrin-positive (perimeter labeled in blue by CellBrite 650 membrane stain, Supplemental Figure 2B) were quantified for comparison. *n*=5 cells with well-spread peripheral membrane per assay×three independent assays=15), * *P* < 0.05 versus *wt+control siRNA or wt+DMSO, ^ P* < 0.05 versus *R218Q+control siRNA or R218Q+DMSO*. (E) Increased total and K48-specific polyubiquitinated proteins in cells with bortezomib treatment shown in immunoblots of ubiquitinated proteins in both cell lysates and GST-S5a UIM-pulldowns. Cell lysates incubated with GST (instead of GST- S5a) served as a control. (F) Percentage of proteasome activity in cells with siRNA-mediated PI31 knockdown (normalized to the mean of cells treated with control siRNA) and in cells pretreated with bortezomib (normalized to the mean of cells treated with DMSO control). CHX, cycloheximide; Co-IP, co-immunoprecipitation; DMSO, dimethyl sulfoxide; Dynll1, dynein light chain 1; GST, glutathione-S-transferase; INF2, inverted formin 2; IP, immunoprecipitation; KI, knockin; MFI, mean fluorescent intensity; PI31, proteasomal inhibitor of 31kD; UIM, ubiquitin-interacting motif; wt, wild-type.

We confirmed the endogenous interaction of Dynll1 and INF2 in *wt/wt* mice by Co-IP from glomerular lysates using an anti-Dynll1 antibody. Minimal PI31 was coimmunoprecipitated with the Dynll1. In the glomerular lysates from R218Q KI mice (both the *wt/ki* and *ki/ki*), there was significantly reduced INF2, but increased PI31, detected in the Dynll1 pulldown, compared with lysates from *wt/wt* glomeruli (quantified as the fractions of INF2 and PI31 proteins in the Dynll1 pulldown relative to the total protein in lysates, Figure 1A). The increased recruitment of PI31 to Dynll1 in the R218Q KI podocytes was confirmed by coimmunostaining of PI31 and Dynll1 (Supplemental Figure 1).

Degradation of nephrin was measured by Western blotting in podocytes cultured in the presence of CHX, which suppressed the synthesis of new proteins. A faster degradation of nephrin was observed in the R218Q KI podocytes than in the *wt* cells, which was largely rescued by siRNA-mediated knockdown of *Dynll1* or *PI31* (Figure 1B), and knockdown of *PI31* did not affect the overall level of proteasomal protease activity (Figure 1F). The INF2 R218Q-accelerated degradation of nephrin was also rescued by pretreating cells with Ciliobrevin D (an inhibitor of dynein) or bortezomib (Figure 1C). The inhibition of proteasomes by bortezomib was demonstrated by the increased level of K48 polyubiquitinated proteins (Figure 1E) and by decreased proteasome protease activity (Figure 1F). Compared with bortezomib, Ciliobrevin D seemed more effective in suppressing nephrin degradation (Figure 1C). We observed reduced nephrin protein, especially at the peripheral membrane of R218Q KI cells, which could also be restored by knockdown of *Dynll1* or *PI31,* by Ciliobrevin D or by bortezomib (Figure 1D and Supplemental Figure 2B).

### Dynein-Induced Mistrafficking and Degradation of Nephrin in R218Q KI Mice Involves the UPS

**Figure 2:**
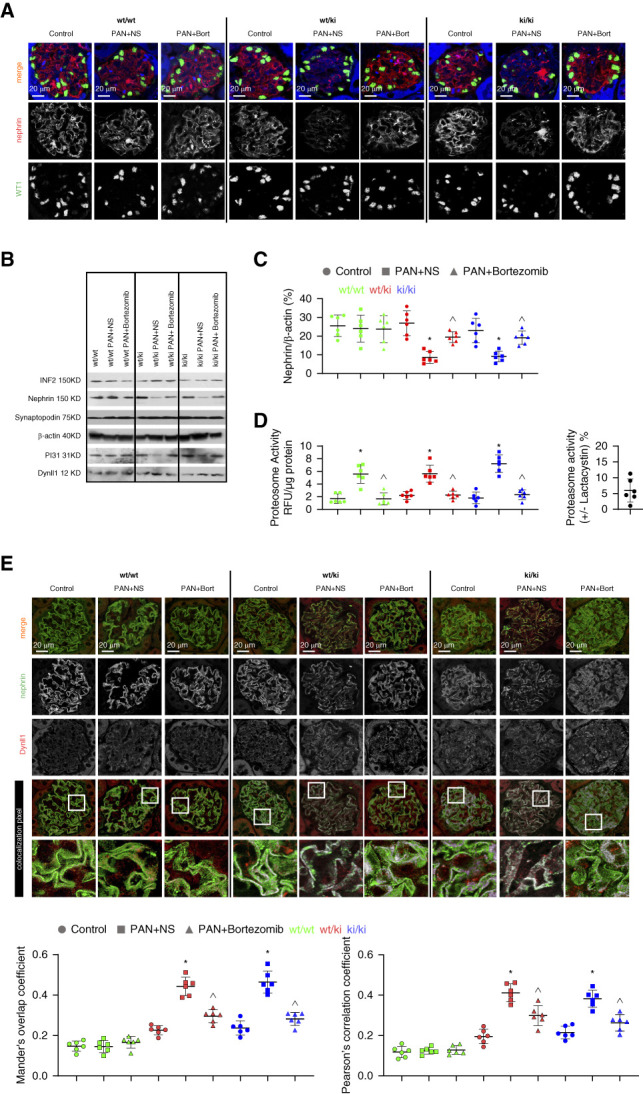
**PA-activated proteasomes deplete nephrin in R218Q KI mice.** (A) Reduced nephrin protein in PAN of R218Q KI mice (*wt/ki and ki/ki* versus *wt/wt*) shown by coimmunofluorescent staining of nephrin (red) and the podocyte marker, WT1 (green). The autofluorescent background was kept in blue channel to show the outline of the mouse kidney histology. (B) Representative Western blots showing the expression of PI31, INF2, Dynll1 and the SD proteins, nephrin and synaptopodin, in mouse glomerular lysates. (C) *β*-actin-normalized nephrin protein in glomerular lysates was compared in mice of different genotypes and treatments (*n*=6 mice per group). (D) Proteasome activity was measured in the glomerular lysates and compared among mice with different genotypes and treatments (*n*=6). The inhibitory effect of 25 *µ*M Lactacystin added into the lysates demonstrated the successful performance of the assay (proteasome activity [+Lactacystin/−Lactacystin]×100%). (E) Coimmunofluorescent staining of nephrin (green) and Dynll1 (red) in kidney sections of control and PA-treated mice of different genotypes (*wt/wt, wt/ki,* and *ki/ki*). Scale bar: 20 *µ*m. By using the Coloc2 plugin of Fiji software, Dynll1-nephrin colocalization was quantified as the Manders overlap coefficient and the Pearson correlation coefficient in five randomly picked glomeruli per mouse kidney section and the mean of measurements per mouse kidney section were calculated for comparison. *n*=6. * *P* < 0.05 versus control mice of the same genotype*, ^ P* < 0.05 versus PA-treated mice+NS treatment control of the same genotype. NS, normal saline; PA, puromycin aminonucleoside; PAN, puromycin aminonucleoside nephropathy; RFU, relative fluorescent units; SD, slit diaphragm; WT1, Wilms tumor 1.

PA challenge increased the activity of proteasomes in the glomeruli of mice (expressed as Proteasome Activity relative fluorescent units per *μ*g protein), regardless of their genotype (Figure 2D). However, PA treatment only resulted in the loss of nephrin protein in R218Q KI mice *(wt/ki* and *ki/ki* versus *wt/wt*), which was rescued by bortezomib treatment (Figure 2, A–C). The homeostasis (as indicated by steady state level) of synaptopodin, another SD protein component, as well as a substrate for proteasome, did not change in the R218Q KI mice with PAN (Figure 2B).

The difference between the *wt/wt* and R218Q KI mice after PA treatment was the enhanced dynein-mediated trafficking of nephrin observed in the latter, reflected by increased colocalization of nephrin and Dynll1 (quantified as the Manders overlap coefficient and as the Pearson correlation coefficient, Figure 2E).

In the R218Q KI mice treated with PA, the expression of Dynll1 remained low. There was a reduced expression of PI31, but the amount of PI31 in the Dynll1 pulldown remained stable (Figure 2B and Supplemental Figure 3).

### Bortezomib Ameliorates PAN in the R218Q KI Mice

**Figure 3:**
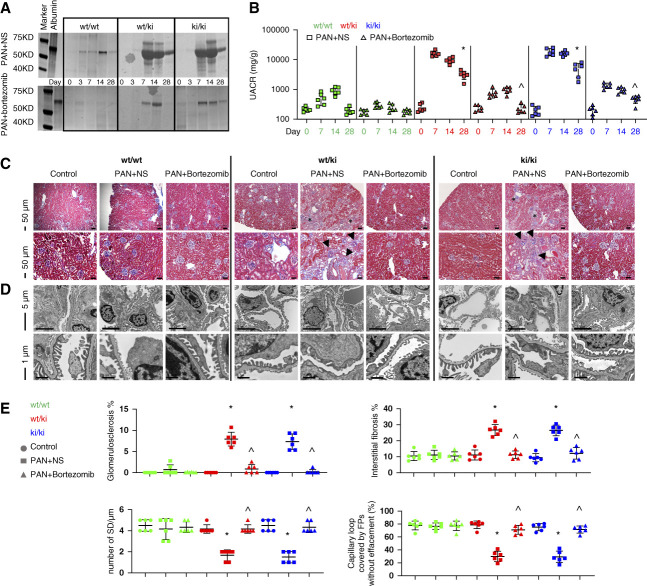
**Bortezomib ameliorated PAN in INF2 R218Q KI mice.** Coomassie blue staining of urine gels (A) and quantification of the UACR, (B) showed the protective effect of bortezomib in ameliorating PA-induced albuminuria in the R218Q KI mice (*wt/ki* and k*i/ki*), compared with NS treatment. The UACR values were compared at the end point of bortezomib treatment (day 28, *n*=6). (C–E) Histological and ultrastructural analyses of kidney sections of wt/wt, wt/ki, and ki/ki mice, each under three treatment conditions: control, PAN+NS, and PAN+Bortezomib, for a total of nine groups (*n*=6 mice per group). Masson Trichrome staining (C, Scale bar: 50 *µ*m), transmission electron microscopy imaging (D), and the histology/ultrastructural quantification (E) of kidney tissue obtained from *wt* and R218Q KI mice with different treatments. (E) The percentage of sclerosed glomeruli (black arrow heads, C); the mean percentage of area with tubulointerstitial fibrosis (asterisks, C) in three nonoverlapping low-power views per mouse kidney section; the average numbers of SDs/*µ*m, and the mean percentage of capillary length covered by foot processes without effacement in three nonoverlapping ultrastructural views per mouse kidney section were compared. *n*=6, * *P* < 0.05 versus control mice of the same genotype*, ^ P* < 0.05 versus PA-treated mice*+*NS treatment control of the same genotype. UACR, urine albumin-to-creatinine ratio.

The *wt/wt* littermates treated with PA had a transient albuminuria that resolved by 1 month. R218Q KI mice (both *wt/ki* and *ki/ki*) challenged with PA showed persistent albuminuria, which could be attenuated by bortezomib treatment, comparing with PA-stressed controls treated with normal saline (NS) injections (Figure 3, A and B).

Compared with the *wt/wt* litter mates, PA induced greater glomerulosclerosis and tubular interstitial fibrosis in the R218Q KI mice, as well as significant podocytopathy, a pathology characterized by foot process effacement, a reduced number of SDs per *µ*m length of glomerular capillaries, and a reduced fraction of capillary loops covered by intact foot processes (Figure 3, C–E). These pathological features of FSGS and podocytopathy were rescued by bortezomib treatment, but not by treatment with normal saline.

## Discussion

Dynein-mediated mistrafficking of nephrin has been found as a pathophysiology of progressive human podocytopathies,^4,17^ especially FSGS caused by the R218Q mutation in the *INF2* gene.^4^ In cultured R218Q KI podocytes, we found an accelerated degradation of nephrin, which could be prevented by bortezomib (a proteasome inhibitor) and Ciliobrevin D (a dynein inhibitor). Compared with bortezomib, Ciliobrevin D seemed more effective in stabilizing nephrin protein in R218Q KI podocytes, suggesting that the UPS is not the sole mode by which nephrin is degraded. Indeed, we had found previously that the ALS can also degrade nephrin mistrafficked by dynein.^4^

To understand how dynein-mediated trafficking subsequently leads to UPS-mediated degradation, we investigated the role of the Dynll1-binding protein, PI31, in connecting the proteasome with the dynein transport complex. By comparing the endogenous interactions between Dynll1 and PI31 in the presence of the wt or R218Q variant of INF2, we demonstrated that, when the Dynll1-INF2 interaction was disrupted by R218Q, the unsequestered Dynll1 recruited more PI31. We then tested the hypothesis that R218Q promotes proteasome-mediated degradation of nephrin by virtue of dynein-proteasome coupling mediated by the Dynll1-PI31 interaction. We showed that the increased nephrin degradation in R218Q KI podocytes could be rescued by knocking down either *Dynll1* or *PI31*, indicating an essential role of Dynll1 and its adaptor PI31 in mediating R218Q-induced nephrin degradation. In parallel, we demonstrated the role of PI31 in impairing the transport of nephrin to the cell surface in R218Q KI podocytes, which could be restored by selective knockdown of PI31. Zaiss *et al*.^20^ observed that overexpression of PI31 in lymphocytes abrogated surface presentation of epitopes in a proteasome-dependent manner and impaired cellular function. Our findings provide evidence of a similar situation at the SDs of podocytes. Although PI31 may facilitate the assembly of the proteasome complex or the recognition of proteasome substrate,^29,30^ we found that the reduced PI31 did not affect total 20S proteasomal protease activity in podocytes, supporting an alternative role of PI31 in connecting dynein cargo with proteasomes. At the SD, tight control of PI31 is required to limit the pathogenic coupling of the UPS with dynein and subsequent degradation of dynein cargoes like nephrin, thereby ensuring an adequate flux of nephrin protein complexes to the cell surface to mediate the filtering function of the glomerular filtration barrier. Accordingly, we were able to restore sorting of nephrin to the cell surface by using *Dynll1* siRNA, or alternatively, by applying bortezomib or Ciliobrevin, probably acting by limiting the degradation of nephrin by a dynein-dependent and UPS-dependent mechanism.

The above findings in cultured R218Q KI podocytes raise the possibility of repurposing the pharmacological proteasome inhibitors (PIs) for the treatment of INF2-related FSGS. Thus, we tested the protective effects of bortezomib in our established model, INF2 R218Q KI mice with PAN, which is a representative *in vivo* model of human R218Q-mediated FSGS.^4^ In R218Q KI mice treated with PA, we hypothesize that the PA interacts with a defect in the podocyte caused by the R218Q mutation, resulting in a more prominent effect than PA with wild-type mice. Compared with the wild-type littermates challenged with PA, the PAN of the R218Q KI mice is characterized by an enhanced dynein-mediated mistrafficking of nephrin, and increased degradation of nephrin through the proteasome pathway, as evidenced by rescue with bortezomib. Subsequently, we found that PA could actually activate proteasomes in glomeruli regardless of the genotype of the mice. The mechanism of how PA activates the proteasome is not clear, but could be explained in principle by the observation that PA upregulates expression of the 26S proteasome and of proteasomal activators, as shown by a transcriptome analysis.^31^ We found that proteasome activation by PA only resulted in a reduction of nephrin protein in the *R218 KI* mice, not in wt siblings. Therefore, the upregulation of the UPS by PA acts in concert with the enhanced dynein-driven mistrafficking of nephrin to proteasomes that is caused by R218Q mutation. By contrast, in the wild-type mice with PA, these joint conditions are not met: PA increases proteasome activity, but the degree of Dynll1-PI31mediated coupling of dynein cargoes to proteasomes is inadequate and enhanced degradation of dynein cargoes through the UPS is prevented. In other words, the degradation of dynein cargoes only happens when they are successfully delivered to proteasomes which are somehow activated, for instance, by PA. This proposed mechanism is supported by the protective effect of bortezomib in the PAN of R218Q KI mice, where an inhibition of the proteasomal proteases broke the pathogenic process, preserved nephrin protein homeostasis, and ameliorated the proteinuric podocytopathy, as well as the development of glomerulosclerosis.

Upregulation of the UPS has been reported in a broader spectrum of kidney diseases, especially those involving profibrotic and NF-*κ*B-related inflammatory pathogenesis.^32,33^ The exposure to profibrotic or proinflammatory events are common but variable factors for the activation of the UPS in adults. Analogous to the role of PA, those profibrotic or inflammatory events may provide a second hit in patients with genetic mutations in INF2 and trigger the clinically heterogeneous INF2-related FSGS. In support of this hypothesis, we have demonstrated that a proteasome inhibitor (PI), bortezomib, could attenuate podocyte injury and prevent glomerulosclerosis in a mouse model of INF2-R218Q-mediated FSGS, by preventing the UPS-mediated depletion of SD proteins. Despite the key role of the UPS in maintaining podocyte health by digesting misfolded proteins,^34,35^ transient use of bortezomib in small-dosing regimens seemed generally tolerated in animal models and human participants, the exception being when it was used in mice that had a concurrent genetic inactivation of the ALS.^36^ Clinically, bortezomib is an US Food and Drug Administration-approved PI that has been used to treat multiple myeloma and associated nephropathy,^37^ with reported success in reducing proteinuria of membranous nephropathy,^38^ antibody-mediated renal rejection,^39^ IgA nephropathy,^40^ and lupus nephritis.^41^ Other drugs like Cyclosporine^42^ and the saquinavir^43^ have been found to possess antiproteinuric effects, which also occur by suppressing the proteasome. While recognizing that modulating proteasome activity in podocytes is at least partially the mechanism of the bortezomib-mediated protection of R218Q-impaired podocytes, we do acknowledge that the consequences of using PIs to target the UPS are broad, with potential side effects that mostly affect the gastrointestinal, cardiovascular, hematological, and neurological systems.^44,45^ In addition, interstitial nephritis has been reported with a newer generation of PI drugs, Carfilzomib.^46^ Some of these side effects could be ameliorated by modifying the dose (peripheral neuropathy, gastroenterological, and hematological side effects) or route of administration.^44^ Some side effects are unique for specific PIs (neurotoxicity of bortezomib versus nephrotoxicity of carfilzomib) while some are related to preexisting conditions or interactions with other medications (*e.g*., with concurrent vincristine treatment^47^). Clinical strategies like tissue-targeting with lipid nanoparticle encapsulation^48,49^ and application of drugs with a favorable safety profile (*e.g*. duloxetine,^50^ antioxidants,^51^ dexanabinol^52^) have shown reassuring protection against these side effects without compromising the therapeutic efficacy of these PIs. While a PI like bortezomib may preserve key podocyte proteins in patients carrying the INF2-R218Q mutation, its constitutive targeting of proteasomes may cause retention of intracellular proteins that harm other normal cells. Therefore, to expand the therapeutic indication of PIs to treat INF2-related podocytopathy, the benefit, risks, preexisting medical conditions, choice of PIs with unique pharmacokinetics and pharmacodynamics need to be taken into consideration. The limitation of PIs underscores the urgency of addressing questions regarding how each individual dynein cargo protein in podocytes is ubiquitylated and selectively sorted to the UPS, and how these processes are altered by INF2 mutations. Understanding these fundamental mechanisms will be necessary to direct the development of more selective PIs with better toxicity profiles and optimized dosing regimens to maximize the therapeutic efficacy while limiting the side effects.

We found this dynein-restricted UPS-dependent proteolysis is more prominent for the homeostasis of nephrin, than for that of synaptopodin, a representative UPS-degraded SD protein that is also important for podocyte morphology and function.^53^ Therefore, the process of R218Q-induced proteolysis in podocytes is likely reserved for a unique pool of dynein cargo proteins (*e.g*., nephrin),^4^ while the homeostasis of many actin-binding SD proteins like synaptopodin is more dependent on Rho small GTPase-mediated actin dynamics.^54^ This distinction suggests that proteolysis through UPS is necessary, but probably not sufficient, for R218Q-induced podocytopathy.

Interestingly, although we observed a reduction of PI31 protein in R218Q KI mice with PAN, we found there was still an adequate amount of PI31 bound to Dynll1, suggesting there is a relative excess of PI31 and that this level is sufficient to capture the low amount of available Dynll1. Therefore, the unsequestrated Dynll1 is probably the limiting factor for the INF2-R218Q-driven degradation of dynein cargoes.

**Figure 4:**
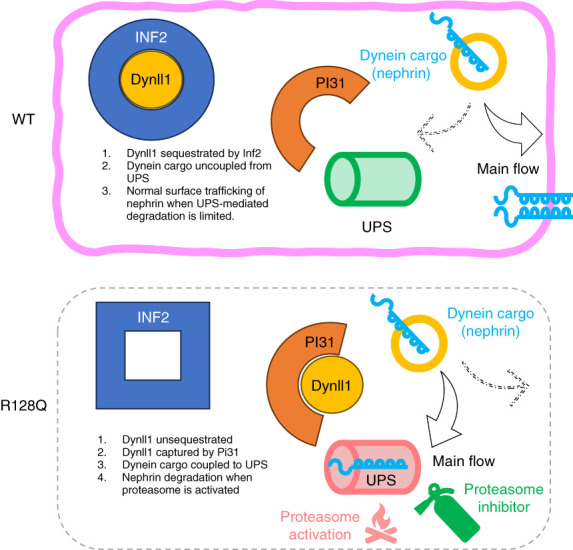
**Schematics.** In R218Q KI podocytes, Dynll1 is not sequestrated by the R218Q variant of INF2, enabling the capture of Dynll1 by PI31, promoting the coupling of the dynein cargo (nephrin) with the proteasome. When the proteasomal proteases are activated by PA, more nephrin is degraded, decreasing the pool of nephrin available for surface trafficking. This pathogenic process can be broken by PIs. In wt podocytes, Dynll1 is sequestered by INF2 and the proteasome-mediated degradation of nephrin is limited, allowing adequate flow of nephrin for functional targeting to the cell surface and subsequent incorporation into SDs. PIs, proteasome inhibitors.

As shown in our schematics (Figure 4), we conclude that the R218Q mutation disrupts the sequestration of Dynll1 by INF2, releasing Dynll1 for recruitment by PI31. Through the Dynll1-coupled adaptor protein PI31, dynein transports nephrin to the proteasome to be degraded, when the UPS is activated (for instance, by PA). In addition to mediating the coupling of dynein cargoes with proteasomes, we speculate that PI31 may boost the function of the UPS through one or more known mechanisms, including facilitating the assembly of 26S proteasome and the recognition of substrate by E3 ligase. However, the lack of obvious nephrin depletion in R218Q KI mice without PA stress indicates these positive regulatory roles of PI31 probably are inadequate to initiate proteolysis through the UPS. However, the positive regulatory effects of PI31 on the UPS potentiate it, making PI31 another candidate molecular target for the future development of proteasome modification therapy.

## Supplementary Material

## Data Availability

All original data in the manuscript are available, including genotype and phenotype data, kidney light, confocal and electron microscopy imaging data of the *INF2* transgenic mice. There are no clinical, genetic, or omics data to declare in our article. The transgenic mice were obtained from Regeneron Pharmaceuticals, under an approved Material Transfer Agreement.

## Supplemental Materials

Supplemental Figure 1. Coimmunofluorescent staining of Dynll1 and PI31.

Supplemental Figure 2. Immunostaining of nephrin and PI31 in podocytes with different treatments and confocal imaging of cells counterstained with CellBrite membrane stain.

Supplemental Figure 3. Dynll1-PI31 interaction in mice with (+) or without (−) PAN.

## References

[1] Andrés-Delgado L Antón OM Madrid R Byrne JA Alonso MA. Formin INF2 regulates MAL-mediated transport of Lck to the plasma membrane of human T lymphocytes. Blood. 2010;116(26):5919–5929. doi:10.1182/blood-2010-08-300665 20881207

[2] Madrid R Aranda JF Rodríguez-Fraticelli AE, . The formin INF2 regulates basolateral-to-apical transcytosis and lumen formation in association with Cdc42 and MAL2. Dev Cell. 2010;18(5):814–827. doi:10.1016/j.devcel.2010.04.001 20493814

[3] Brown EJ Schlöndorff JS Becker DJ, . Mutations in the formin gene INF2 cause focal segmental glomerulosclerosis. Nat Genet. 2010;42(1):72–76. doi:10.1038/ng.505 20023659 PMC2980844

[4] Sun H Perez-Gill C Schlondorff JS Subramanian B Pollak MR. Dysregulated dynein-mediated trafficking of nephrin causes INF2-related podocytopathy. J Am Soc Nephrol. 2021;32(2):307–322. doi:10.1681/ASN.2020081109 33443052 PMC8054882

[5] Morales-Alvarez MC Knob A Rennke HG Pollak MR Denker BM. Clinical and pathological heterogeneity in FSGS due to INF2 mutations. Kidney Int Rep. 2022;7(12):2741–2745. doi:10.1016/j.ekir.2022.08.033 36506246 PMC9727514

[6] Barua M Brown EJ Charoonratana VT Genovese G Sun H Pollak MR. Mutations in the INF2 gene account for a significant proportion of familial but not sporadic focal and segmental glomerulosclerosis. Kidney Int. 2013;83(2):316–322. doi:10.1038/ki.2012.349 23014460 PMC3647680

[7] Boyer O Benoit G Gribouval O, . Mutations in INF2 are a major cause of autosomal dominant focal segmental glomerulosclerosis. J Am Soc Nephrol. 2011;22(2):239–245. doi:10.1681/ASN.2010050518 21258034 PMC3029896

[8] Sun H Schlondorff JS Brown EJ Higgs HN Pollak MR. Rho activation of mDia formins is modulated by an interaction with inverted formin 2 (INF2). Proc Natl Acad Sci U S A. 2011;108(7):2933–2938. doi:10.1073/pnas.1017010108 21278336 PMC3041098

[9] Sun H Schlondorff J Higgs HN Pollak MR. Inverted formin 2 regulates actin dynamics by antagonizing Rho/diaphanous-related formin signaling. J Am Soc Nephrol. 2013;24(6):917–929. doi:10.1681/ASN.2012080834 23620398 PMC3665394

[10] Sun H Al-Romaih KI MacRae CA Pollak MR. Human kidney disease-causing INF2 mutations perturb Rho/Dia signaling in the glomerulus. EBioMedicine. 2014;1(2-3):107–115. doi:10.1016/j.ebiom.2014.11.009 26086034 PMC4457406

[11] Subramanian B Sun H Yan P, . Mice with mutant Inf2 show impaired podocyte and slit diaphragm integrity in response to protamine-induced kidney injury. Kidney Int. 2016;90(2):363–372. doi:10.1016/j.kint.2016.04.020 27350175 PMC5363079

[12] Subramanian B Chun J Perez-Gill C, . FSGS-causing INF2 mutation impairs cleaved INF2 N-fragment functions in podocytes. J Am Soc Nephrol. 2020;31(2):374–391. doi:10.1681/ASN.2019050443 31924668 PMC7003299

[13] Slaats GG Braun F Hoehne M, . Urine-derived cells: a promising diagnostic tool in Fabry disease patients. Sci Rep. 2018;8(1):11042. doi:10.1038/s41598-018-29240-w 30038331 PMC6056427

[14] Meyer-Schwesinger C. Lysosome function in glomerular health and disease. Cell Tissue Res. 2021;385(2):371–392. doi:10.1007/s00441-020-03375-7 33433692 PMC8523507

[15] Heintz L Meyer-Schwesinger C. The intertwining of autophagy and the ubiquitin proteasome system in podocyte (Patho)Physiology. Cell Physiol Biochem. 2021;55(S4):68–95. doi:10.33594/000000432 34523304

[16] Yoshida S Wei X Zhang G, . Endoplasmic reticulum-associated degradation is required for nephrin maturation and kidney glomerular filtration function. J Clin Invest. 2021;131(7):e143988. doi:10.1172/JCI143988 33591954 PMC8011890

[17] Sun H Weidner J Allamargot C Piper RC Misurac J Nester C. Dynein-mediated trafficking: a new mechanism of diabetic podocytopathy. Kidney360. 2023;4(2):162–176. doi:10.34067/KID.0006852022 36821608 PMC10103215

[18] Liu K Jones S Minis A Rodriguez J Molina H Steller H. PI31 is an adaptor protein for proteasome transport in axons and required for synaptic development. Dev Cell. 2019;50(4):509–524.e10. doi:10.1016/j.devcel.2019.06.009 31327739 PMC6702053

[19] Li X Thompson D Kumar B DeMartino GN. Molecular and cellular roles of PI31 (PSMF1) protein in regulation of proteasome function. J Biol Chem. 2014;289(25):17392–17405. doi:10.1074/jbc.M114.561183 24770418 PMC4067172

[20] Zaiss DM Standera S Kloetzel PM Sijts AJ. PI31 is a modulator of proteasome formation and antigen processing. Proc Natl Acad Sci U S A. 2002;99(22):14344–14349. doi:10.1073/pnas.212257299 12374861 PMC137886

[21] Buneeva OA Kopylov AT Medvedev AE. Proteasome interactome and its role in the mechanisms of brain plasticity. Biochemistry (Mosc). 2023;88(3):319–336. doi:10.1134/S0006297923030033 37076280

[22] Keir LS Firth R May C Ni L Welsh GI Saleem MA. Generating conditionally immortalised podocyte cell lines from wild-type mice. Nephron. 2015;129(2):128–136. doi:10.1159/000369816 25720381

[23] Buchanan BW Lloyd ME Engle SM Rubenstein EM. Cycloheximide chase analysis of protein degradation in Saccharomyces cerevisiae. J Visualized Experiments. 2016(110):53975. doi:10.3791/53975 PMC4941941 27167179

[24] Rabe M Schaefer F. Non-transgenic mouse models of kidney disease. Nephron. 2016;133(1):53–61. doi:10.1159/000445171 27212380

[25] Shimo T Adachi Y Yamanouchi S, . A novel nuclear factor κB inhibitor, dehydroxymethylepoxyquinomicin, ameliorates puromycin aminonucleoside-induced nephrosis in mice. Am J Nephrol. 2013;37(4):302–309. doi:10.1159/000348803 23548793

[26] Zeniya M Mori T Yui N, . The proteasome inhibitor bortezomib attenuates renal fibrosis in mice via the suppression of TGF-β1. Sci Rep. 2017;7(1):13086. doi:10.1038/s41598-017-13486-x 29026167 PMC5638992

[27] Hainz N Thomas S Neubert K, . The proteasome inhibitor bortezomib prevents lupus nephritis in the NZB/W F1 mouse model by preservation of glomerular and tubulointerstitial architecture. Nephron Exp Nephrol. 2012;120(2):e47–e58. doi:10.1159/000334955 22286140

[28] Liu X Fan Q Yang G, . Isolating glomeruli from mice: a practical approach for beginners. Exp Ther Med. 2013;5(5):1322–1326. doi:10.3892/etm.2013.1000 23737872 PMC3671739

[29] Bader M Benjamin S Wapinski OL Smith DM Goldberg AL Steller H. A conserved F box regulatory complex controls proteasome activity in Drosophila. Cell. 2011;145(3):371–382. doi:10.1016/j.cell.2011.03.021 21529711 PMC3108249

[30] Minis A Rodriguez JA Levin A, . The proteasome regulator PI31 is required for protein homeostasis, synapse maintenance, and neuronal survival in mice. Proc Natl Acad Sci U S A. 2019;116(49):24639–24650. doi:10.1073/pnas.1911921116 31754024 PMC6900516

[31] Beeken M Lindenmeyer MT Blattner SM, . Alterations in the ubiquitin proteasome system in persistent but not reversible proteinuric diseases. J Am Soc Nephrol. 2014;25(11):2511–2525. doi:10.1681/ASN.2013050522 24722446 PMC4214514

[32] Bonni S Wang HR Causing CG, . TGF-beta induces assembly of a Smad2-Smurf2 ubiquitin ligase complex that targets SnoN for degradation. Nat Cell Biol. 2001;3(6):587–595. doi:10.1038/35078562 11389444

[33] Coppo R. Proteasome inhibitors in progressive renal diseases. Nephrol Dial Transplant. 2014;29(suppl 1):i25–i30. doi:10.1093/ndt/gft271 24493867

[34] Makino SI Shirata N Oliva Trejo JA, . Impairment of proteasome function in podocytes leads to CKD. J Am Soc Nephrol. 2021;32(3):597–613. doi:10.1681/ASN.2019101025 33510039 PMC7920174

[35] Radon V Czesla M Reichelt J, . Ubiquitin C-Terminal Hydrolase L1 is required for regulated protein degradation through the ubiquitin proteasome system in kidney. Kidney Int. 2018;93(1):110–127. doi:10.1016/j.kint.2017.05.016 28754552

[36] Hartleben B Godel M Meyer-Schwesinger C, . Autophagy influences glomerular disease susceptibility and maintains podocyte homeostasis in aging mice. J Clin Invest. 2010;120(4):1084–1096. doi:10.1172/JCI39492 20200449 PMC2846040

[37] Burnette BL Leung N Rajkumar SV. Renal improvement in myeloma with bortezomib plus plasma exchange. N Engl J Med. 2011;364(24):2365–2366. doi:10.1056/NEJMc1101834 21675906

[38] Geara AS Bhoj V Hogan JJ. Bortezomib treatment for refractory PLA2R-positive membranous nephropathy. Glomerular Dis. 2021;1(1):40–43. doi:10.1159/000515087 36751489 PMC9677711

[39] Waiser J Budde K Schutz M, . Comparison between bortezomib and rituximab in the treatment of antibody-mediated renal allograft rejection. Nephrol Dial Transplant. 2012;27(3):1246–1251. doi:10.1093/ndt/gfr465 21852274

[40] Hartono C Chung M Perlman AS, . Bortezomib for reduction of proteinuria in IgA nephropathy. Kidney Int Rep. 2018;3(4):861–866. doi:10.1016/j.ekir.2018.03.001 29988921 PMC6035125

[41] Segarra A Arredondo KV Jaramillo J, . Efficacy and safety of bortezomib in refractory lupus nephritis: a single-center experience. Lupus. 2020;29(2):118–125. doi:10.1177/0961203319896018 31865857

[42] Meyer S Kohler NG Joly A. Cyclosporine A is an uncompetitive inhibitor of proteasome activity and prevents NF-kappaB activation. FEBS Lett. 1997;413(2):354–358. doi:10.1016/s0014-5793(97)00930-7 9280312

[43] Coppo R Camilla R Porcellini MG, . Saquinavir in steroid-dependent and -resistant nephrotic syndrome: a pilot study. Nephrol Dial Transplant. 2012;27(5):1902–1910. doi:10.1093/ndt/gfs035 22431705

[44] Pancheri E Guglielmi V Wilczynski GM, . Non-hematologic toxicity of bortezomib in multiple myeloma: the neuromuscular and cardiovascular adverse effects. Cancers (Basel) 2020;12(9):2540. doi:10.3390/cancers12092540 32906684 PMC7563977

[45] Gavriatopoulou M Malandrakis P Ntanasis-Stathopoulos I Dimopoulos MA. Nonselective proteasome inhibitors in multiple myeloma and future perspectives. Expert Opin Pharmacother. 2022;23(3):335–347. doi:10.1080/14656566.2021.1999411 34761710

[46] Efentakis P Lamprou S Makridakis M, . Mineralocorticoid receptor pathway is a key mediator of carfilzomib-induced nephrotoxicity: preventive role of eplerenone. Hemasphere. 2022;6(11):e791. doi:10.1097/HS9.0000000000000791 36285072 PMC9584194

[47] Geisler S. Vincristine- and bortezomib-induced neuropathies - from bedside to bench and back. Exp Neurol. 2021;336:113519. doi:10.1016/j.expneurol.2020.113519 33129841 PMC11160556

[48] Maksimos M Muz B Magnani JL Azab AK. E-selectin-targeting lipid nanoparticles improve therapeutic efficacy and reduce side effects of bortezomib in multiple myeloma. Blood Cancer J. 2023;13(1):48. doi:10.1038/s41408-023-00828-4 37029121 PMC10081993

[49] Dutta D Liu J Wen K, . BCMA-targeted bortezomib nanotherapy improves therapeutic efficacy, overcomes resistance, and modulates the immune microenvironment in multiple myeloma. Blood Cancer J. 2023;13(1):184. doi:10.1038/s41408-023-00955-y 38072962 PMC10711001

[50] Mansooralavi N Khomula EV Levine JD. Duloxetine prevents bortezomib and paclitaxel large-fiber chemotherapy-induced peripheral neuropathy (LF-CIPN) in sprague dawley rats. Mol Pain. 2023;19:17448069231185694. doi:10.1177/17448069231185694 37338165 PMC10288414

[51] Iijima Y Bandow K Amano S, . Protection of bortezomib-induced neurotoxicity by antioxidants. Anticancer Res. 2020;40(7):3685–3696. doi:10.21873/anticanres.14357 32620607

[52] Bloomingdale P Meregalli C Pollard K, . Systems pharmacology modeling identifies a novel treatment strategy for bortezomib-induced neuropathic pain. Front Pharmacol. 2021;12:817236. doi:10.3389/fphar.2021.817236 35126148 PMC8809372

[53] Robins R Baldwin C Aoudjit L Gupta IR Takano T. Loss of Rho-GDIα sensitizes podocytes to lipopolysaccharide-mediated injury. Am J Physiol Ren Physiol. 2015;308(11):F1207–F1216. doi:10.1152/ajprenal.00225.2014 25503727

[54] Ning L Suleiman HY Miner JH. Synaptopodin is dispensable for normal podocyte homeostasis but is protective in the context of acute podocyte injury. J Am Soc Nephrol. 2020;31(12):2815–2832. doi:10.1681/ASN.2020050572 32938649 PMC7790210

