# Supplementary material for: Dynll1-PI31 Interaction Enhances Proteolysis Through the Proteasome, Representing a Novel Therapeutic Target for INF2-Related FSGS
Source: Kidney360. 2024 Dec 2;6(1):38–48. doi: 10.34067/KID.0000000659 (PMC11793186; doi:10.34067/KID.0000000659)
Supplement: Supplementary file 1 [file kidney360-6-038-s001.pdf]

ASN American Society of Nephrology | JASN Journal of the American Society of Nephrology | KIDNEY360 Accessing Our World From Every Angle | CJASN Clinical Journal of the American Society of Nephrology

ASN Journal Disclosure Form

As per ASN journal policy, I have disclosed any financial relationships or commitments I have held in the past 36 months as included below. I have listed my Current Employer below to indicate there is a relationship requiring disclosure. If no relationship exists, my Current Employer is not listed.

C. Allamargot reports the following:
Employer: The University of Iowa

I understand that the information above will be published within the journal article, if accepted, and that failure to comply and/or to accurately and completely report the potential financial conflicts of interest could lead to the following: 1) Prior to publication, article rejection, or 2) Post-publication, sanctions ranging from, but not limited to, issuing a correction, reporting the inaccurate information to the authors' institution, banning authors from submitting work to ASN journals for varying lengths of time, and/or retraction of the published work.

Name: Chantal Allamargot
Manuscript ID: K360-2024-000469R2
Manuscript Title: Dynll1-PI31 interaction enhances proteolysis via the proteasome, representing a novel therapeutic target for INF2-related FSGS
Date of Completion: November 15, 2024
Disclosure Updated Date: November 15, 2024

ASN Journal Disclosure Form

As per ASN journal policy, I have disclosed any financial relationships or commitments I have held in the past 36 months as included below. I have listed my Current Employer below to indicate there is a relationship requiring disclosure. If no relationship exists, my Current Employer is not listed.

C. Perez-Gill reports the following:
Employer: University of Colorado Anschutz Medical Campus

I understand that the information above will be published within the journal article, if accepted, and that failure to comply and/or to accurately and completely report the potential financial conflicts of interest could lead to the following: 1) Prior to publication, article rejection, or 2) Post-publication, sanctions ranging from, but not limited to, issuing a correction, reporting the inaccurate information to the authors' institution, banning authors from submitting work to ASN journals for varying lengths of time, and/or retraction of the published work.

Name: Chandra Perez-Gill
Manuscript ID: K360-2024-000469R1
Manuscript Title: Dynll1-PI31 interaction enhances proteolysis via the proteasome, representing a novel therapeutic target for INF2-related FSGS
Date of Completion: September 3, 2024
Disclosure Updated Date: September 3, 2024

ASN American Society of Nephrology | JASN Journal of the American Society of Nephrology | KIDNEY360 Accessing Our World From Every Angle | CJASN Clinical Journal of the American Society of Nephrology

ASN Journal Disclosure Form

As per ASN journal policy, I have disclosed any financial relationships or commitments I have held in the past 36 months as included below. I have listed my Current Employer below to indicate there is a relationship requiring disclosure. If no relationship exists, my Current Employer is not listed.

M. Pollak reports the following:
Employer: Beth Israel Deaconess Medical Center; Harvard Medical School; Consultancy: Vertex; Research Funding: Vertex; Honoraria: Various academic talks; Patents or Royalties: Athena Diagnostics; Advisory or Leadership Role: NephCure Foundation scientific advisory board; and Other Interests or Relationships: Scientific Advisory Board, NephCure Foundation.

I understand that the information above will be published within the journal article, if accepted, and that failure to comply and/or to accurately and completely report the potential financial conflicts of interest could lead to the following: 1) Prior to publication, article rejection, or 2) Post-publication, sanctions ranging from, but not limited to, issuing a correction, reporting the inaccurate information to the authors' institution, banning authors from submitting work to ASN journals for varying lengths of time, and/or retraction of the published work.

Name: Martin Pollak
Manuscript ID: K360-2024-000469R2
Manuscript Title: Dynll1-PI31 interaction enhances proteolysis via the proteasome, representing a novel therapeutic target for INF2-related FSGS
Date of Completion: November 5, 2024
Disclosure Updated Date: November 5, 2024

ASN American Society of Nephrology
JASN Journal of the American Society of Nephrology
KIDNEY360 Accessing Our World From Every Angle
CJASN Clinical Journal of the American Society of Nephrology

ASN Journal Disclosure Form

As per ASN journal policy, I have disclosed any financial relationships or commitments I have held in the past 36 months as included below. I have listed my Current Employer below to indicate there is a relationship requiring disclosure. If no relationship exists, my Current Employer is not listed.

F. Rooney reports the following:
Employer: University of Iowa

I understand that the information above will be published within the journal article, if accepted, and that failure to comply and/or to accurately and completely report the potential financial conflicts of interest could lead to the following: 1) Prior to publication, article rejection, or 2) Post-publication, sanctions ranging from, but not limited to, issuing a correction, reporting the inaccurate information to the authors' institution, banning authors from submitting work to ASN journals for varying lengths of time, and/or retraction of the published work.

Name: Faith F Rooney
Manuscript ID: K360-2024-000469R1
Manuscript Title: Dynll1-PI31 interaction enhances proteolysis via the proteasome, representing a novel therapeutic target for INF2-related FSGS
Date of Completion: September 3, 2024
Disclosure Updated Date: September 3, 2024

ASN American Society of Nephrology | JASN Journal of the American Society of Nephrology | KIDNEY360 Accessing Our World From Every Angle | CJASN Clinical Journal of the American Society of Nephrology

ASN Journal Disclosure Form

As per ASN journal policy, I have disclosed any financial relationships or commitments I have held in the past 36 months as included below. I have listed my Current Employer below to indicate there is a relationship requiring disclosure. If no relationship exists, my Current Employer is not listed.

H. Sun reports the following:
Employer: University of Iowa; and Research Funding: NIH 1R01DK136563 (PI: Sun), 5K12HD027748 (PI: Bassuk), University of Iowa Sted Family Children's Hospital Children's Miricle fund (CMN86023539).

I understand that the information above will be published within the journal article, if accepted, and that failure to comply and/or to accurately and completely report the potential financial conflicts of interest could lead to the following: 1) Prior to publication, article rejection, or 2) Post-publication, sanctions ranging from, but not limited to, issuing a correction, reporting the inaccurate information to the authors' institution, banning authors from submitting work to ASN journals for varying lengths of time, and/or retraction of the published work.

Name: Hua Sun
Manuscript ID: K360-2024-000469R1
Manuscript Title: Dynll1-PI31 interaction enhances proteolysis via the proteasome, representing a novel therapeutic target for INF2-related FSGS
Date of Completion: September 3, 2024
Disclosure Updated Date: September 3, 2024

ASN
American Society of Nephrology
JASN
Journal of the American Society of Nephrology
KIDNEY360
Accessing Our World From Every Angle
CJASN
Clinical Journal of the American Society of Nephrology

ASN Journal Disclosure Form

As per ASN journal policy, I have disclosed any financial relationships or commitments I have held in the past 36 months as included below. I have listed my Current Employer below to indicate there is a relationship requiring disclosure. If no relationship exists, my Current Employer is not listed.

J. Williquett reports the following:
Employer: The University of Iowa

I understand that the information above will be published within the journal article, if accepted, and that failure to comply and/or to accurately and completely report the potential financial conflicts of interest could lead to the following: 1) Prior to publication, article rejection, or 2) Post-publication, sanctions ranging from, but not limited to, issuing a correction, reporting the inaccurate information to the authors' institution, banning authors from submitting work to ASN journals for varying lengths of time, and/or retraction of the published work.

Name: Jillian Williquett
Manuscript ID: K360-2024-000469R2
Manuscript Title: Dynll1-PI31 interaction enhances proteolysis via the proteasome, representing a novel therapeutic target for INF2-related FSGS
Date of Completion: October 1, 2024
Disclosure Updated Date: September 3, 2024
