# Supplementary material for: Dynll1-PI31 Interaction Enhances Proteolysis Through the Proteasome, Representing a Novel Therapeutic Target for INF2-Related FSGS
Source: Kidney360. 2024 Dec 2;6(1):38–48. doi: 10.34067/KID.0000000659 (PMC11793186; doi:10.34067/KID.0000000659)
Supplement: Supplementary file 2 [file kidney360-6-038-s002.pdf]

Supplementary Figures

Table of contents

Supplementary methods.

Supplementary figure 1. Co-immunofluorescent staining of Dynll1 and PI31.

Supplementary figure 2. Immunostaining of nephrin and PI31 in podocytes with different treatments and confocal imaging of cells counterstained with CellBrite membrane stain.

Supplementary figure 3. Dynll1-PI31 interaction in mice with (+) or without (–) puromycin aminonucleoside nephropathy (PAN)

Supplementary methods

Immunofluorescent staining: Cells fixed in 4% paraformaldehyde were processed for co-immunofluorescent staining for nephrin (Alexa Fluor 594-conjugated mouse anti-nephrin) and PI31 (rabbit anti-PI31 followed by Alexa Fluor 488-conjugated anti-Rabbit IgG, Table 1). The amount of nephrin protein was quantified as the mean fluorescence intensity (MFI) per podocyte; the nephrin distribution at the edge of the cell (labeled by CellBrite® Steady Membrane Stain, Biotium) was quantified as the fraction of perimeter membrane with positive nephrin staining.

K48 Polyubiquitin Chain Capture assay: Cells lysates were prepared in RIPA buffer containing 2 mM 2-Mercaptoethanol, 5 mM Iodoacetamide (IAA) and cOmplete, Mini Protease Inhibitor Cocktail (Roche). Following the manufacturer's instructions (K48 Polyubiquitin Chain Capture assay kit, UBP bio, Table 1), cell lysates containing 5 mg of protein were incubated with 0.2mg of either glutathione S-transferase (GST) (negative control) or GST-S5a (Ubiquitin-Interacting Motif [UIM], a homolog of the ubiquitin binding S5a subunit of the 26S proteasome), precipitated using glutathione resin and eluted with a buffer containing 10 mM glutathione. The captured K48 polyubiquitinated proteins were assayed by immunoblotting using mouse anti-ubiquitin (Santa Cruz).

Proteasome activity assay: Following the manufacturer's instructions (20S Proteasome Activity Assay kit, Millipore Sigma APT280),[1] mouse glomeruli or cultured podocytes were lysed in lysis buffer (50 mM HEPES (pH 7.5), 5 mM EDTA, 150 mM NaCl and 1% Triton X-100). The lysates were centrifuged at 15,000 rpm for 15 minutes at 4°C, and the supernatants were harvested for

assay. 2 mM ATP was added to the lysates to improve the recovery of intact 26S proteasome. As an assay control, some lysates were incubated with 25μM Lactacystin (a 20S proteasome inhibitor provided in the assay kit) at room temperature for 15 minutes. Lysates were loaded in duplicate into black 96-well plates with the substrate mix provided by the kit and incubated at 37 °C for 60 min. The fluorescence was then read in a SpectraMax Spectrofluorometer using 380 nm excitation and 460 nm emission settings and divided by total protein loaded per well and expressed as relative fluorescent units (RFU) per μg protein. Inhibition of proteasome activity by Lactacystin were expressed as the percentage ($RFU^{+Lactacystin}$/$RFU^{-Lactacystin}$) x100%.

Co-Immunoprecipitation and western blotting: Mouse glomeruli were lysed on ice for 1 hour in Nonidet P40 lysis buffer with 0.5% deoxycholate supplemented with cOmplete, Mini Protease Inhibitor Cocktail (Roche), and PhosSTOP phosphatase inhibitor cocktail (Sigma). Pooled glomerular lysates from 2 mice of the same group were immunoprecipitated with rabbit anti-Dynll1 (Thermo Fisher # PA5-97920), using normal rabbit IgG as control. The immunoprecipitated proteins were separated by SDS-PAGE and transferred to polyvinylidene fluoride or polyvinylidene difluoride membranes. After blocking, western blotting was then performed to detect the Inf2 and PI31 proteins in the Dynll1-pulldown, using rabbit anti-INF2 (Bethyl Lab # A303–427A) and rabbit ant-PI31 (Sigma # HPA041300), respectively. For quantification, densitometry was performed using Fiji software.

References

1. Osburn SC, Vann CG, Church DD, Ferrando AA, Roberts MD. Proteasome- and Calpain-Mediated Proteolysis, but Not Autophagy, Is Required for Leucine-Induced Protein Synthesis in C2C12 Myotubes. *Physiologia*. Dec 2021;1(1):22-33. doi:10.3390/physiologia1010005

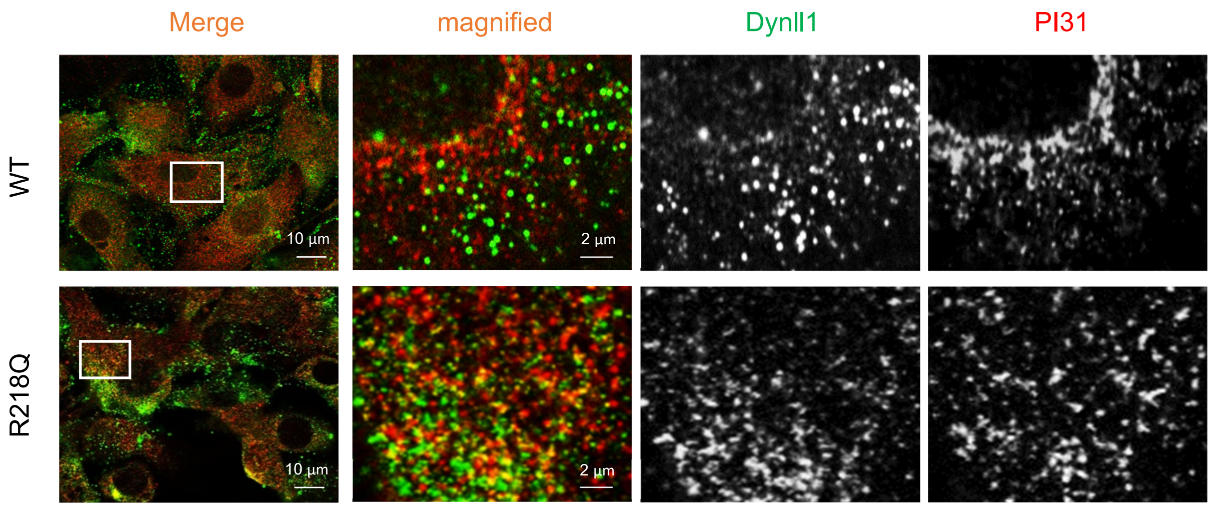

Supplementary figure 1. Co-immunofluorescent staining of Dynll1 and PI31.

Immunofluorescent staining showed increased recruitment of PI31 (red) to Dynll1 (green) in R218Q KI podocytes, compared to wildtype podocytes. Scale bars= 10µm (2µm in magnified figures).

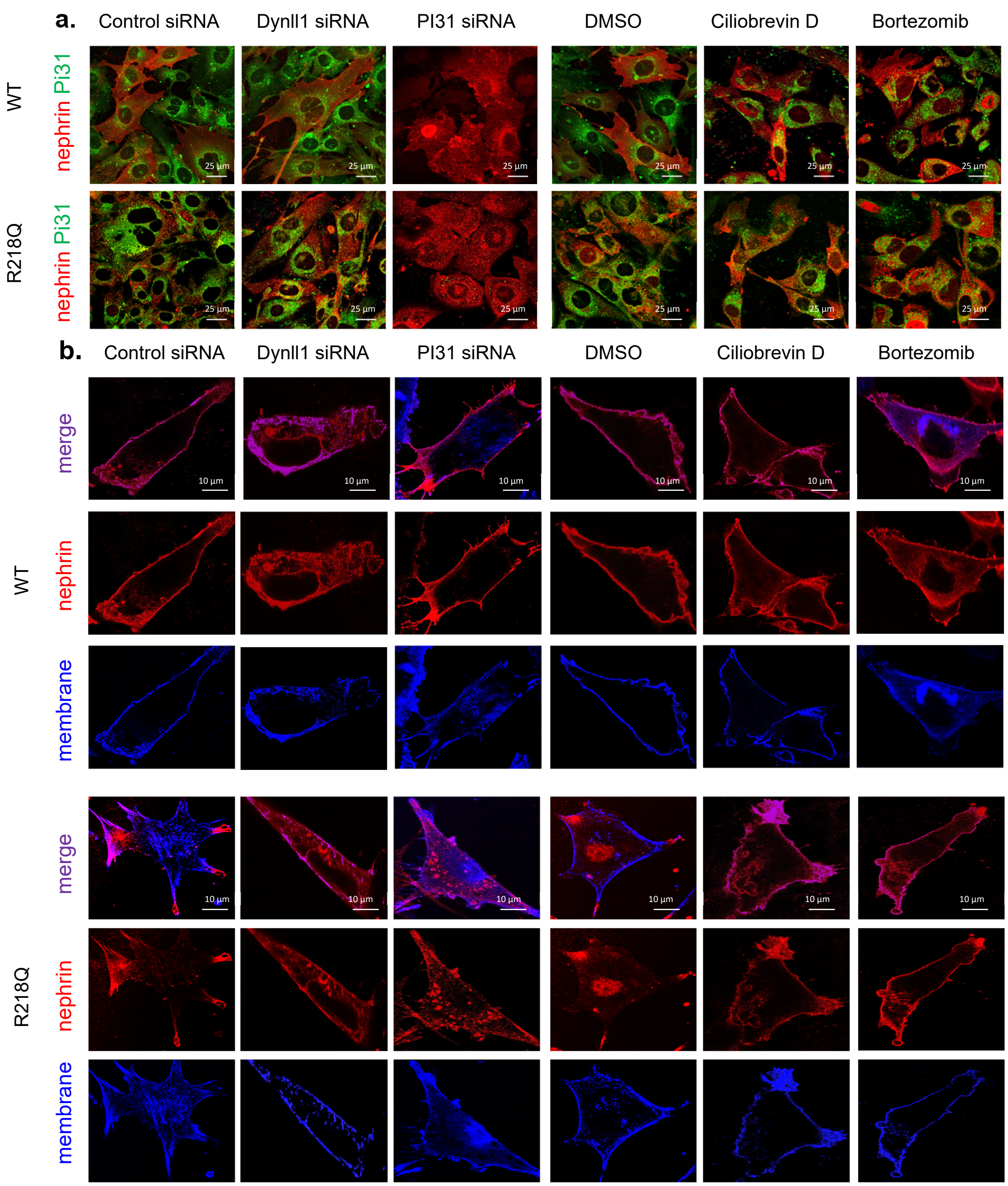

Supplementary figure 2. Immunostaining of nephrin and PI31 in podocytes with different treatments and confocal imaging of cells counterstained with CellBrite membrane stain.

a. Lower power view (scale bar=25 µm) of immunofluorescent staining of nephrin (red) and PI31 (green) in WT and R218Q KI podocytes with different treatments. Cells with siRNA-mediated knockdown of *Dynll1* or *PI31 were* compared to cells transfected with control siRNA. Cells treated with Ciliobrevin D (50 µM) or bortezomib (100 nM) were compared to cells treated with vehicle (0.3% DMSO). **b.** Confocal microscopic imaging of representative cells with well-spread peripheral membrane. Cells were co-stained with nephrin (red) and CellBrite Steady 650 Membrane Stain (blue). The percentage of nephrin-positive peripheral membrane (labeled by CellBrite membrane stain) was quantified in Image J for comparison.

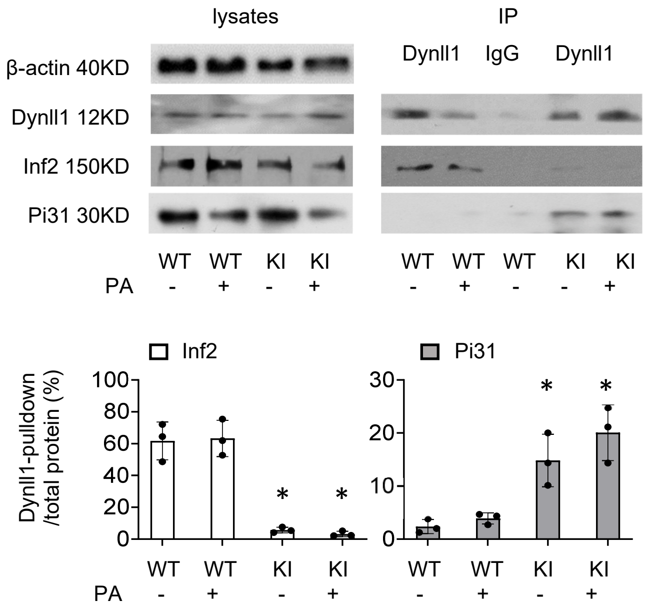

Supplementary figure 3. Dynll1-PI31 interaction in mice with (+) or without (–) puromycin aminonucleoside nephropathy (PAN)

As examined by Co-IP, the amount of PI31 in the Dynll1-pulldown remained stable in glomerular lysates of wt/wt (WT) and *R218Q* ki/ki (KI) mice following puromycin aminonucleoside treatment (PA). n=3, * *$p<0.05$ vs. wt/wt (without PA).*
